# COVID-19 susceptibility: potential of *ACE2* polymorphisms

**DOI:** 10.1186/s43042-020-00099-9

**Published:** 2020-09-21

**Authors:** Mayank Chaudhary

**Affiliations:** Department of Biotechnology, Maharishi Markandeshwar (Deemed to be University), Mullana, Ambala, Haryana 133207 India

**Keywords:** Single-nucleotide polymorphism (SNP), ACE2, Severe acute respiratory syndrome (SARS), COVID-19, SARS-CoV-2, Coronavirus

## Abstract

**Background:**

Angiotensin-converting enzyme 2 (ACE2) is a metallopeptidase that primarily functions as a negative regulator of renin angiotensin system (RAS) by converting angiotensin II (Ang II) to angiotensin 1-7. Contrary to this, another RAS component, angiotensin-converting enzyme (ACE) catalyzes synthesis of Ang II from angiotensin I (Ang I) that functions as active compound in blood pressure regulation. This indicates importance of ACE/ACE2 level in regulating blood pressure by targeting Ang II. An outbreak of severe acute respiratory syndrome (SARS) highlighted the additional role of ACE2 as a receptor for SARS coronavirus (SARS-CoV) infection.

**Main body of the abstract:**

ACE2 is a functional receptor for SARS-CoV and SARS-CoV-2. Activation of spike (S)-protein by either type II transmembrane serine proteases (TTSPs) or cathepsin-mediated cleavage initiates receptor binding and viral entry. In addition to TTSPs, ACE2 can also be trimmed by ADAM 17 (a disintegrin and metalloproteinase 17) that competes for the same receptor. Cleavage by TTSPs activates ACE2 receptor for binding, whereas ADAM17 releases extracellular fragment called soluble ACE2 (sACE2). Structural studies of both ACE2 and S-protein have found critical sites involved in binding mechanism. In addition to studies on structural motifs, few single-nucleotide polymorphism (SNPs) studies have been done to find an association between genetic variants and SARS susceptibility. Though no association was found in those reports, but seeing the non-reproducibility of SNP studies among different ethnic groups, screening of *ACE2* SNPs in individual population can be undertaken.

**Short conclusion:**

Thus, screening for novel SNPs focussing on recently identified critical regions of ACE2 can be targeted to monitor **s**usceptibility towards coronavirus disease 2019 (COVID-19).

## Background

Angiotensin-converting enzyme (ACE) is a critical component of renin angiotensin system (RAS) that is involved in blood pressure homeostasis [1]. Components of RAS are expressed both systemically and in tissue specific manner [2]. Cleavage of angiotensin I (Ang I) by ACE results in generation of angiotensin II (Ang II) that binds to either angiotensin II type 1 receptor (AT1R) or angiotensin II type 2 receptor (AT2R) [3]. Binding of Ang II to AT1R is mainly involved in blood pressure regulation. Contrary to this, another homolog of ACE, ACE2 encoded by human X-chromosome can either act on Ang I to give rise to angiotensin 1-9 [4] or on Ang II to generate angiotensin 1-7 [5]. These newly synthesized products from Ang II acts on the Mas receptor that is expressed in tissues related to cardiovascular disease. Binding of Ang II products to Mas receptor can lower blood pressure through vasodilation or through sodium and water excretion in addition to nitric oxide production [6]. In this way, ACE2 negatively regulates ACE-Ang II signaling effects that primarily cause an increase in blood pressure.

Another major role of ACE2 was highlighted by the emergence of severe acute respiratory syndrome (SARS) in 2002–2003 caused by SARS coronavirus (SARS-CoV) [7, 8]. Human ACE2 acted as functional receptor and binds to spike (S) protein of SARS-CoV with high affinity [9]. Localization of ACE2 protein in various human tissues was explored and was found to be abundantly present in epithelia of lung and intestine, providing a possible route of entry to SARS-CoV [10]. Presence of ACE2 in human airway epithelia was also studied and abundant expression was seen in well-differentiated epithelial cells. In addition to this, predominant expression was seen in apical than the basolateral surface which suggests the availability of enzyme for cleavage of peptides at mucosal surfaces of airway [11]. Similar result was found by Xu et al. [12] where ACE2 expression was observed on the mucosa of the oral cavity.

Coronaviruses are divided into α, β, γ, and δ genera on the basis of the target host. Out of these, mammals are infected by α and β-CoV whereas γ and δ-CoV genera tend to infect birds [13]. A recent respiratory tract infection that has taken the form of pandemic by the name of COVID-19 (coronavirus disease 2019) is found to be caused by SARS-CoV-2 [14]. It is a β-CoV that is enveloped, non-segmented and positive-sense RNA virus [15]. Genome sequencing results showed that this newly discovered virus share 96.2% identity with bat CoV RaTG13 and 79.5% identity with SARS-CoV. This data suspected bat to be the natural host and transmission to humans via unknown intermediate host [16]. Recent metagenomic study considered pangolins (*Manis javanica*) as possible intermediate host that might have acquired the SARS-CoV-2 virus from bats. This consideration was based on putative recombination signals between coronavirus of pangolin, bat, and human [17]. Dependence of this newly discovered virus on the ACE2 receptor to infect humans has again drawn the attention of entire scientific communities towards the binding mechanism of this receptor with viral protein which is considered as a critical step for the entry of the virus. Binding efficiency of S-protein of SARS-CoV-2 with ACE2 was found to be higher than SARS-CoV [18]. Readers interested to know about origin, epidemiology, genome structure, transmission, clinical characteristics, and present medication against SARS-CoV-2 are recommended for Guo et al. [13] as the current article focuses on recent findings within binding sites of ACE2 and SARS-CoV.

**Fig. 1:**
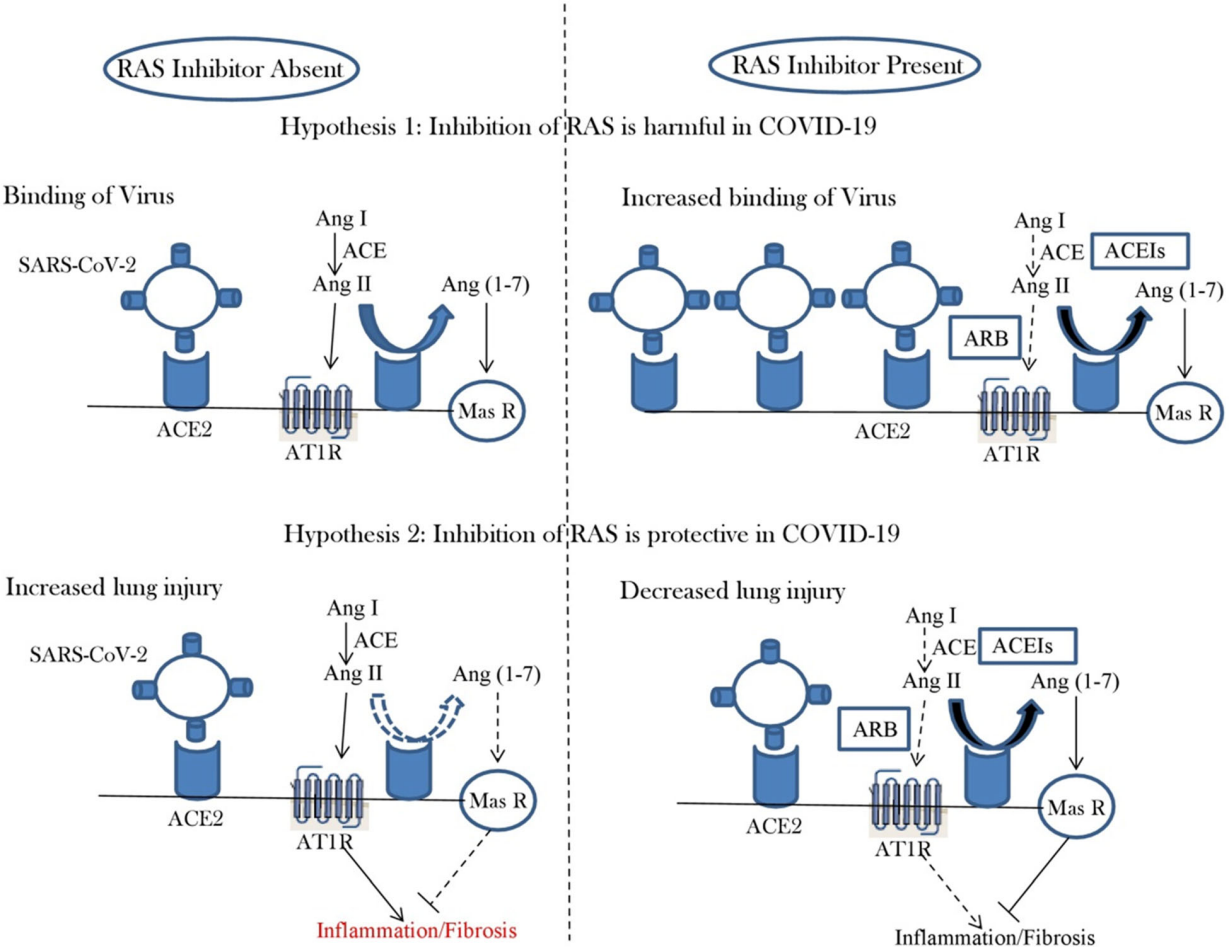
Possible relation between renin-angiotensin system inhibition and COVID-19 [adapted from 19]. Competing hypothetical mechanisms by which use of ACEIs and ARBs might be harmful or protective in COVID-19. Hypothesis 1: SARS-CoV-2 enters cell by binding to ACE2. Use of ACEIs and ARBs could increase ACE2 abundance and enhance viral entry. Hypothesis 2: Ang II causes lung injury through inflammation and fibrosis upon activation of AT1R. Reduced production of Ang II by ACEIs or blockade of Ang II-AT1R interaction by ARBs increases generation of Ang (1-7) by ACE2 and activates Mas receptor (MasR) causing reduction in inflammation and fibrosis and thereby attenuating lung injury

Dual functionality of ACE2 as blood pressure regulator and as a receptor for binding of virus particles initiated a debate on the susceptibility of hypertensive individuals against the current pandemic of COVID-19 that are undergoing anti-hypertensive treatment with ACE inhibitors (ACEIs) or angiotensin receptor blockers (ARBs). Different hypothesis have been given (Fig. 1) suggesting the effect of anti-hypertensive medication on viral binding and lung injury [19]. Results in animal models showed upregulation of *ACE2* in heart and kidney upon treatment with ACEIs and ARBs [20, 21]. These findings raised concerns on the susceptibility of patients for severity of COVID-19 that are undergoing similar anti-hypertensive treatment. But studies showing similar effect of ACEIs and ARBs on expression of *ACE2* in the lungs are lacking. Moreover, other studies have not reported any such role of ACEIs and ARBs on the expression pattern of *ACE2* [22, 23]. In addition to this, experimental models have suggested that blockade of AT1R though ARBs can reduce Ang II-mediated acute lung injury [24]. Reduction in lung injury can further result in weakening of COVID-19 infection. Therefore, no such human study is available at present which supports the hypothesis that usage of ACEIs and ARBs increases the risk of SARS-CoV-2 infection [19]. This is further supported by the fact that various international societies working on hypertension have recommended continuation of ACEIs and ARBs due to absence of convincing evidence against these medications in present scenario.

## Main text

### ACE2 as functional receptor for SARS-CoV

**Fig. 2:**
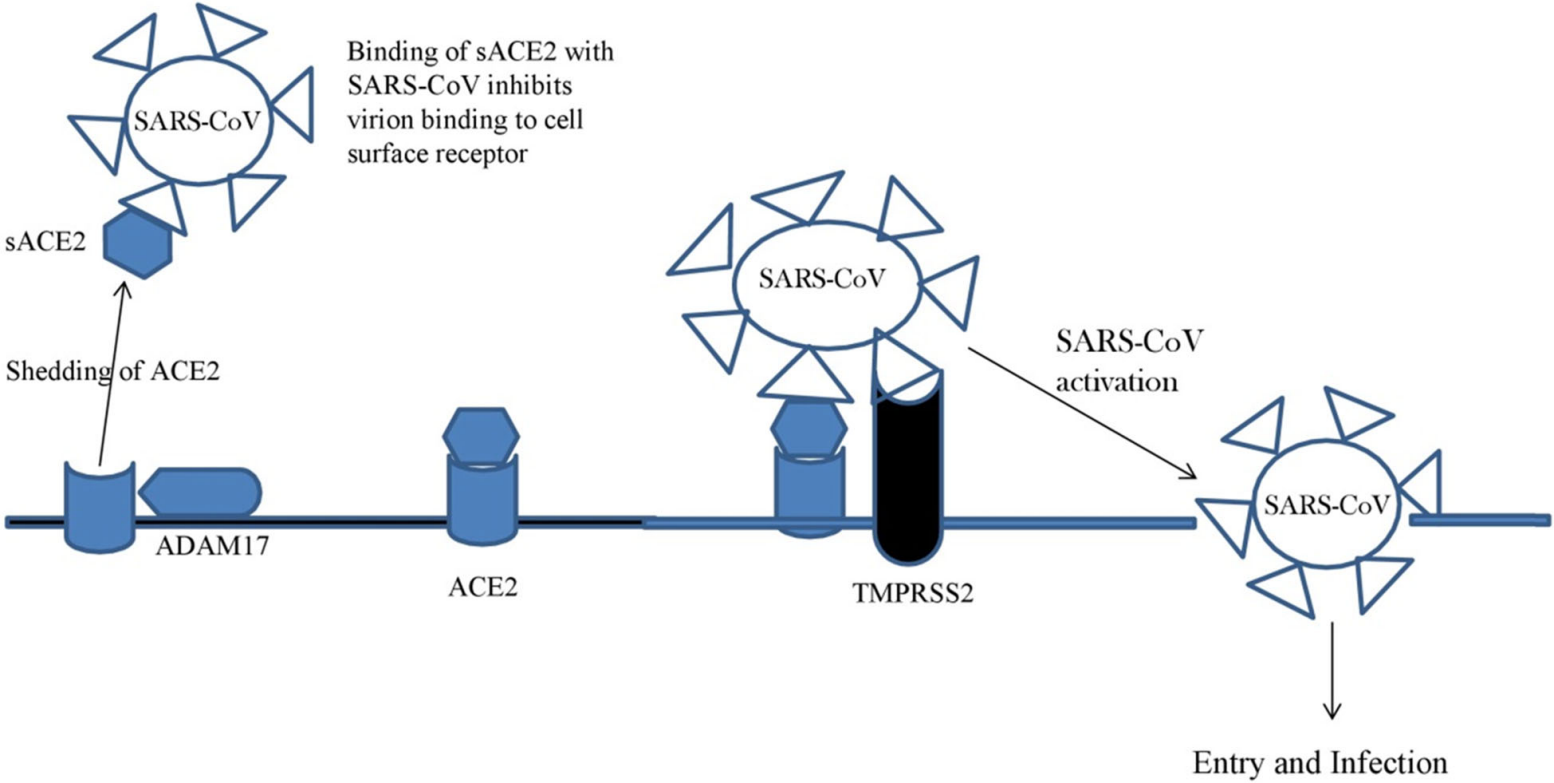
Action of host cell proteases on ACE2 receptor [adapted from 35]. Cleavage of ACE2 by ADAM17 causes its shedding. Interaction of sACE2 with S-Protein of SARS-CoV prevents binding of virus particles to target cells. Co-expression of TMPRSS2 with ACE2 on target cell surface involves binding of SARS-CoV (S-protein) to ACE2- and TMPRSS2-mediated processing allows fusion and uptake of virus particles

ACE2 protein is a transmembrane glycoprotein comprised of 805 amino acids with extracellular catalytic domain, small transmembrane fragment, and short C-terminal cytoplasmic tail [25]. The spike (S) protein of SARS-CoV mediates entry by binding to ACE2 receptor present on cell surface followed by fusion of the viral envelope with host cell membrane [26]. S protein consists of S1 and S2 subunits where S1 subunit is responsible for receptor binding and S2 is responsible for membrane fusion [27]. S1 subunit contains the receptor-binding domain (RBD) at residues 318-510 [28] and S2 harbor elements that are responsible for membrane fusion. Binding of SARS-S protein to ACE2 initiates conformational change in SARS-S that increases proteolytic cleavage of S protein [29]. Activation of spike (S) protein of SARS-CoV involves cleavage by cathepsin [30] or by type II transmembrane serine proteases (TTSPs) that include transmembrane protease serine 2 (TMPRSS2) and human airway trypsin-like protease (HAT) [31]. Both TTSPs are co-expressed with ACE2 in human lung cells [32]. ACE2 can be proteolytically processed by serine proteases (TMPRSS2 and HAT) resulting in SARS-CoV entry or by ADAM17 (a disintegrin and metalloproteinase 17) resulting in the release of extracellular fragment called soluble ACE2 (sACE2) [25, 33]. The role of ADAM17 in the shedding of ACE2 was experimentally proved by modulating ADAM17 expression [34]. Overexpression of ADAM17 increased ACE2 shedding whereas application of natural ADAM inhibitors reduced shedding of ACE2. In addition to this, both TMPRSS2 and ADAM17 compete for cleavage of ACE2 [35] (Fig. 2) but both these proteases have different cleavage sites. TTSPs dependent proteolysis requires the presence of arginine and lysine residues within 697-716 amino acids, whereas ADAM17 requires the presence of arginine and lysine residues within 652-659 amino acids for cleavage of ACE2. As the observed cleavage site of ADAM17 differed from previous studies [36, 37] so the identified sites were proposed as probable recognition sites for downstream cleavage. Synthesis of sACE2 by ADAM17-mediated processing (Fig. 2) retains its enzymatic activity and can inhibit binding of SARS-S to target cells [36]. These results were in accordance of study done by Li et al. [9] where the soluble form of ACE2 (sACE2) blocked association between the S1 domain and cultured cells transfected with ACE2. As the binding mechanism of SARS-CoV-2 is similar to SARS-CoV so the synthesis of cyclodextrin (CD) and sACE2 complex is suggested as a suitable methodology to block SARS-CoV-2 infection [25]. In addition to this, certain studies have found important regions within receptor binding domain (RBD) of the S1 subunit of SARS-S protein that can be targeted. Deletion of the positively charged region (422-463 amino acids) of RBD affected virus infectivity as an amino acid substitution at targeted sites (R441A) and (R453A) abolished viral entry [38]. Similarly, an important hexapeptide (Tyr-Lys-Tyr-Arg-Tyr-Leu) at 438-443 amino acids was found in RBD [39]. In addition to structural studies on RBD, significant amino acid residues of ACE2 protein at other sites have also been discovered. The amino acid residues at position 31, 41, 353, 355, and 357 of ACE2 were found to be significant [27, 40] as mutation at these sites strongly inhibited interaction of ACE2 with SARS-S protein. Comparison of rhesus (rh-ACE2) and human (hu-ACE2) yielded non-synonymous substitution and generation of hu-ACE2 (Y217N) mutant caused significant reduction in protein expression and viral entry [41].

### Single-nucleotide polymorphisms (SNPs) in *ACE2*

Single-nucleotide polymorphism studies have been done in different populations to find association with various diseases. SNPs have been found to affect gene expression resulting in disease outcome. However, these association studies are greatly affected by factors like ethnicity, age, and selection criteria that result in controversial results among different population groups. SNPs of *ACE2* have also been studied in different populations to find association with diseases, including essential hypertension, dyslipidemia, hypertrophic cardiomyopathy, ventricular hypertrophy, and cerebral malaria [42–44]. Many of the studied polymorphisms were found to affect ACE2 activity resulting in downregulation of circulating angiotensin (1-7). Association studies targeting *ACE2* polymorphisms have reported controversial results in different populations which clearly show the non-reproducibility of association studies. Our literature search targeting *ACE2* polymorphisms with severe acute respiratory syndrome resulted in two specific studies [45, 46] other than recent reports on COVID-19. Through software based search Chiu et al. [45] identified 103 SNPs in *ACE2* which included 2 coding (rs4646116, rs4646179) and 101 intronic SNPs. SNP validation confirmed sequence variation at only 5 non-coding SNP loci (rs2106809, rs2285666, rs4646142, rs714205, rs2074192). These 5 SNPs were screened in a case-control study involving SARS patients and healthy volunteers, but no statistically significant difference of any of the studied SNPs was found [45]. Thus, no association was found between genetic variants and SARS susceptibility. Similarly, the association of *ACE2* SNPs with SARS was studied in Vietnam population where each exon and 5′UTR region was screened for SNPs [46]. This study identified 19 SNPs from which 13 novel and two other SNPs (rs2285666, rs183135788) were screened among collected samples, but no significant difference was found in genotypic and allelic frequencies of these SNPs.

**Fig. 3:**
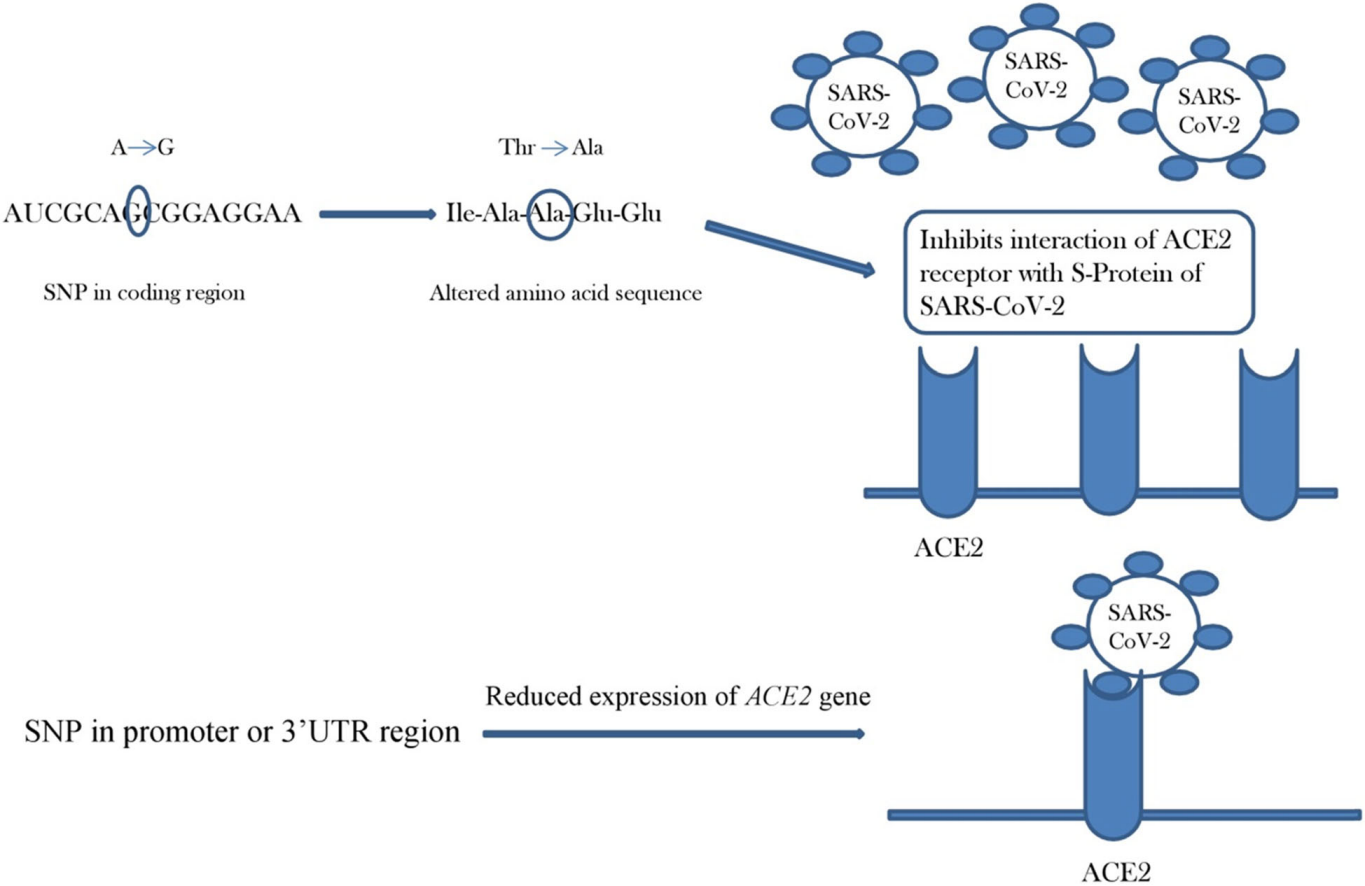
Possible role of novel *ACE2* SNPs in susceptibility towards SARS-CoV-2. Presence of SNPs within coding region can result in alteration of amino acid sequence. This change in amino acid sequence at site of interaction can affect the binding capacity of ACE2 receptor with S-Protein of SARS-CoV-2. Novel SNPs within promoter or 3′-UTR can cause downregulation of *ACE2* gene resulting in lower levels of ACE2 receptor at cell surface for interaction with virus particles

Lack of association of any functional *ACE2* polymorphism with SARS infection might be due to lesser number of association studies done previously. Moreover, seeing the possibility of contradictions that arise from such SNP studies among different populations, role of *ACE2* variation in susceptibility to SARS infection cannot be ruled out. This hypothesis gets support from a previous study where variation in HIV co-receptor developed resistance to HIV infection in the Caucasian population [47]. Similarly, a recent study has identified four polymorphisms in type II transmembrane protein dipeptidyl peptidase 4 (DPP4) that greatly reduced binding and penetration of the middle-east respiratory syndrome coronavirus (MERS-CoV) into target cells [48]. These facts highlight the importance of SNP variation within the identified crucial binding sites [27, 41] and at amino acid residues responsible for either TMPRSS2- or ADAM17-mediated proteolysis of ACE2 [35]. This idea gets support from other studies also where change in key amino acids responsible for interaction is considered crucial for cross-species infections, multi-host infection, and differences in disease susceptibility [17]. Screening of these sites along with promoter regions in different populations can result in identification of novel SNPs that might affect susceptibility to SARS infection (Fig. 3). Analysis of the upstream region of *ACE2* gene showed the bipartite nature of *ACE2* promoter with the presence of two promoter regions separated by an Alu element. These promoters result in distal promoter transcripts (DPT) and proximal promoter transcripts (PPT) that encode same ACE2 protein. Both proximal and distal ACE2 promoter regions possess hepatocyte nuclear factor 1α (HNF1 α) binding motifs that induce *ACE2* expression in pancreatic islets [49]. Thus, both distal and proximal promoter regions should be considered while screening for novel SNPs within *ACE2* promoter region.

Results from comparative analysis of ACE2 orthologs among 70 placental mammal species showed 85% similarity between pangolin and human ACE2. Out of the total variable sites, 30 sites relevant for interaction with SARS-CoV were screened for interspecies variation. Additionally, same sites were also studied for intraspecific variation in humans. These sites were highly conserved within *Homo sapiens* and showed great variation among placental mammalian species [17]. Recent reports have hypothesized correlation between ACE2 levels and susceptibility to present infection. Higher expression of *ACE2* was reported in lungs of men than women. This was related to the severity of the disease in case of males. Moreover, Asian population showed higher *ACE2* expression than Caucasian and African American population [50]. Contrary to this, the role of estrogen in upregulation of *ACE2* expression and plasma ACE2 activity was suggested as a possible reason for protection of females against COVID-19 infection in comparison to males [33]. Genetic analysis of expression quantitative trait loci (eQTL) and functional coding variants in *ACE2* was done to identify mutations resistant to the binding of coronavirus S-protein in different populations [51]. Though no such mutations were identified, but difference in allelic frequencies of certain coding SNPs and eQTL variants were found between Chinese and European population. Higher allelic frequency of certain variants resulted in higher *ACE2* expression in Chinese population. These findings highlighted association of *ACE2* polymorphism with higher ACE2 expression in East Asian population. Thus, difference in *ACE2* expression levels was suggested to cause differential susceptibility for SARS-CoV-2 among different populations under similar conditions. In this context, Wooster et al. [52] identified *ACE2* polymorphisms that might influence disease severity. Out of 10 studied SNPs, 5 polymorphisms (rs4240157, rs6632680, rs4830965, rs1476524, and rs2048683) showed an association with higher tissue specific expression of ACE2 resulting in hospitalization whereas rs1548474 polymorphism showed association with low tissue expression and lesser severity. Similarly, variation in circulating ACE2 levels was speculated to be controlled by genetic factors where rs2106809 polymorphism might affect ACE2 levels. CC or CT genotype resulted in greater circulating ACE2 levels when compared with TT genotype. Therefore, quantification of human soluble ACE2 (sACE2) in body fluids was suggested as a protective biomarker for rapid test screening. In addition to a possible link between circulating ACE2 levels and severity of disease, availability of recombinant ACE2 is considered as a hopeful treatment option [33]. Thus, difference in either tissue specific ACE2 expression or plasma ACE2 levels can affect disease severity. This signifies the importance of identifying novel SNPs that can either affect tissue specific ACE2 expression or plasma ACE2 levels. Other studies have also highlighted an association of AC*E2* and *TMPRSS2* polymorphisms with COVID-19 susceptibility and infection [53, 54]. Additionally, coronavirus infection is related to the state of hypercytokinemia/cytokine storm which is characterized by an excessive synthesis of pro-inflammatory cytokines resulting in severe outcomes that might include multiple organ damage. Such response of hypercytokinemia was seen in ACE2-positive cells. So, genetic polymorphism in genes responsible for the synthesis of pro-inflammatory cytokines and chemokines along with *ACE2* might be responsible for differences in response to COVID-19 [55]. Link between ACE2 levels and immune response can also be related from a study where reduced promoter methylation caused higher expression of *ACE2* resulting in immune infiltration of certain tumor cells [56]. As till date, no effective medicine or vaccine is present against COVID-19 so identification of any such functional SNP that can affect population susceptibility will be of great help.

## Conclusion

The role of ACE2 in addition to blood pressure regulation was highlighted from emergence of severe acute respiratory syndrome (SARS) in 2002–2003. Recent outbreak of COVID-19 again highlighted the functional role of ACE 2 as a receptor for the spike (S) protein of SARS-CoV-2 to mediate viral entry. With the availability of no medicine/vaccine at present, the binding potential of ACE2 with S protein along with the role of serine proteases in activation of S protein is vastly explored. In addition to these mechanisms, screening for novel SNPs in recently identified crucial regions of ACE2 can also be targeted for studying susceptibility towards current pandemic.

## Data Availability

There is no availability of data and materials.

## Abbreviations

ACE: Angiotensin-converting enzyme
ACE2: Angiotensin-converting enzyme 2
ACEIs: Angiotensin-converting enzyme inhibitors
ADAM17: A disintegrin and metalloproteinase 17
Ang I: Angiotensin I
Ang II: Angiotensin II
ARBs: Angiotensin receptor blockers
AT1R: Angiotensin II type 1 receptor
AT2R: Angiotensin II type 2 receptor
CD: Cyclodextrin
COVID-19: Coronavirus disease 2019
DPP4: Dipeptidyl peptidase 4
DPT: Distal promoter transcripts
eQTL: Expression quantitative trait loci
HAT: Human airway trypsin-like protease
HNF1 α: Hepatocyte nuclear factor 1α
MERS-CoV: Middle-east respiratory syndrome coronavirus
PPT: Proximal promoter transcripts
RAS: Renin angiotensin system
RBD: Receptor-binding domain
S protein: Spike protein
sACE2: Soluble ACE2
SARS: Severe acute respiratory syndrome
SARS-CoV: SARS coronavirus
SNP: Single-nucleotide polymorphism
TMPRSS2: Transmembrane protease serine 2
TTSPs: Type II transmembrane serine proteases
